# Comment on Cary Moskovitz’ “Text Recycling in Health Sciences Literature: A Rhetorical Perspective”

**DOI:** 10.1186/s41073-017-0026-y

**Published:** 2017-02-16

**Authors:** Miguel Roig

**Affiliations:** Department of Psychology, St. John’s University, Staten Island, NY USA

**Keywords:** Text recycling, Plagiarism, Self-plagiarism, Paraphrasing, Publication ethics

## Abstract

The question of covert text recycling from previous publications is discussed. It is argued that, consistent with current guidance, authors may be allowed to covertly recycle a limited amount of their previously published material but mainly at the phrase level and only when it is composed of very complex descriptions laden with technical terms for which there are no suitable substitutes. Authors may recycle longer segments of text using standard scholarly conventions of quotation and attribution or via some other informal means that alerts readers as to the scope of the recycling, thereby ensuring transparency. The use of percent similarity scores as thresholds for acceptable amounts of reuse should be discouraged. Instead, editors should be given the flexibility to evaluate each instance of recycling by taking into account factors such as the technical nature of the recycled text and the language proficiency of the authors.

This article is a response to the following commentary: http://researchintegrityjournal.biomedcentral.com/articles/10.1186/s41073-017-0025-z.

## Main text

In discussing the problem of text recycling (i.e., authors’ covert reuse of their previously disseminated text), Moskovitz [1] identifies two issues that he feels ought to be taken into consideration: That citation and attribution practices differ across disciplines and that the different needs of editors and readers should be acknowledged and taken into account. In the spirit of continuing the dialogue, I would like to comment on both of these themes.

Before doing so, I wish to clarify that the on-line document on avoiding plagiarism [2] that Moskovitz cites and which I first created in 2003 with financial support from the US Office of Research Integrity (ORI) was written with the aim to “help students, as well as professionals, identify and prevent *questionable practices* (my emphasis) and to develop an awareness of ethical writing” (page 1). The document was designed to be consistent with guidance offered by many other sources created to help students and professionals write scholarly and scientific papers. It is also important to emphasize that a review of ORI’s definition of misconduct and, specifically, of its definition of plagiarism [3] will fail to yield any reference to the notion of self-plagiarism, of which text recycling is a form of. However, ORI has acknowledged that many forms of self-plagiarism are in violation of most journals’ submission policies, but these transgressions generally fail to meet ORI’s definition of misconduct unless they involve possible falsification of data [4]. As Moskovitz’ paper [1] demonstrates, text recycling continues at best to be considered as a questionable practice by some. Thus, it seems to me that advising prospective authors to use conservative interpretations of traditional scholarly practices of quotation and attribution represents sound guidance in the context of the stated purpose of the document. Given the wide-ranging opinions on the matter, to suggest differently falls short of what would be considered a “best practice” approach to scientific scholarship. Perhaps most importantly, at a time when the public’s trust in science continues to diminish and calls for transparency in all aspects of the scientific process are becoming louder, the emphasis on training should be on practices that encourage openness and excellence, not on practices that many deem as questionable.

The above position notwithstanding, few can deny Moskovitz’ point that minor text recycling in the health sciences is unavoidable and that in some cases it may be outright desirable, a point that I have made in the older version of the plagiarism resource, [2] in its more recent version [5] and elsewhere [6]. Where we seem to disagree is in the extent to which such reuse should be permissible and in the conditions that should allow it. For example, Moskovitz quotes from the BioMed Central Guidelines on text recycling [7], which, essentially, state that some recycling at the level of phrases may be permissible in some sections of the IMRAD-type paper (i.e., Introduction-Method-Results-And-Discussion papers). This is a reasonable position and one that is consistent with the guidelines provided in the plagiarism resource [2, 5]. But, then he quotes from an editorial in the *Canadian Journal of Hospital Pharmacy* [8] to illustrate how others hold the practice to be “intrinsically problematic”. However, a closer read of the portion of the editorial quoted by Moskovitz shows that its author is against repeating passages verbatim, not necessarily against repeating phrases. The distinction is crucial because the word “passages” denotes longer portions of text, such as entire sentences or even paragraphs. It is the recycling of these larger portions of text that many of us in the scientific community find most objectionable, especially when the recycled content is not terribly technical and can be reasonably restated without fear that doing so would compromise its message. As I point out in my instructional resource [2, 5]:

“ … methodology sections often include very intricately complex descriptions of procedural processes that are laden with unique terminology and phraseology for which there are no acceptable equivalents (e.g., *Mammalian histone lysine methyltransferase, suppressor of variegation 39H1 (SUV39H1*). Even when major textual modifications to these sections are possible, a change in the language can run the risk of slightly altering the intended meaning of what is being described and such an outcome is a highly undesirable in the sciences. Thus authors should be allowed some latitude in terms of the extent to which they should modify portions of text when paraphrasing material from methodology sections that is highly technical in nature, even if the material is derived from other sources” (page 24).

So, especially when it comes to technical material and perhaps not limited to methods sections, not only should some limited reuse of one’s own previously published technical text be allowed at the level of phrases, but also similar small portions of text from others’ published works. Consider what ORI’s definition of plagiarism [3] states about the latter point: “ORI generally does not pursue the limited use of identical or nearly-identical phrases which describe a commonly-used methodology or previous research because ORI does not consider such use as substantially misleading to the reader or of great significance”. Thus, based on ORI’s definition of plagiarism, citation and attribution in the sciences is somewhat different from that of the humanities, but the difference is minimal and within the very narrow scope of phrases that describe complex, technical materials or processes.

Moskovitz points to the needs of readers and editors and, with respect to the former group, alludes to the benefits of being able to read the exact message again. Specifically, he complains that paraphrasing “accurate and perfectly effective prose” for purposes of avoiding a charge of self-plagiarism results in “superficial and arbitrary changes that ultimately make reading harder for those following the line of research”. One assumption with this line of thinking is that what is conveyed in published papers is so well-written that it can never benefit from additional clarification, elaboration, or even a complete restatement of the message. I agree that rewriting earlier published prose merely for purposes of changing its appearance and avoiding a charge of self-plagiarism is not what we want to encourage authors to do. Instead, the emphasis should be on convincing authors of the benefits of improving their message’s clarity and on using new language to increase its probability of a more enduring impact on readers. Yes, repeating the previous message verbatim may make it easier for readers to recognize the ideas being conveyed, but long-standing evidence from memory research shows that changing the structure and language of a message has its unique benefits also. Indeed, a thorough paraphrase of previously published material may, under certain conditions, lead readers to struggle a bit more to assimilate and accommodate that same message in their current conceptual schema, but evidence from the Levels of Processing model of memory [9] indicates that a good paraphrase will increase the chances that readers will have a better understanding of, and better odds of recalling, those ideas [10, 11].

One aspect of many discussions of plagiarism, text recycling, and related malpractices is that it often neglects a constituent group whose needs ought to be acknowledged perhaps to a greater extent than the needs of editors and readers: Authors who are not native speakers of English and likely not very proficient in that language. Keeping in mind that English has become the official language of science and that an increasing proportion of the scientific literature is authored by researchers whose dominant language is not English [12], is it any wonder that a significant amount of text recycling of their own and of others’ work occurs amongst this particular group of authors? [13, 14] To be fair, plagiarism and other writing and publication infractions are also committed by native English speakers. Moreover, some of those who defend text recycling [15, 16] may, in fact, have been raised and educated with English as their mother tongue. But a relevant question in the context of English’ dominance of the scientific literature is why it is that we are having a conversation about text recycling today and not, say, 50 years ago. One reason should be obvious to those who have been following the research integrity literature and, specifically, the emerging literature on publication ethics. It seems that in the rush to increase their publication output, too many authors, non-native as well as native speakers of English, have been “stretching” what once may have been the collective consensus of what ought to be an acceptable form and amount of text recycling. Thus, we seem to have moved from the unavoidable reuse of a few highly technical phrases, such as those for which no substitutes are available, to the abusive recycling of large portions of text leading to corrections and/or retractions of journal articles [17, 18].

Another factor that likely plays a more fundamental role in our urge to reuse our previously published text is the fact that for many authors, writing does not come easy regardless of their level of English proficiency. In addition to needing to acquire the unique vocabulary and conceptual frameworks of a scientific discipline, authors must also develop the art of concise and clear writing that is expected in an IMRAD-type of publication. Thus, acquiring a nominal level of competency in scientific writing can take much time, great mental effort, and considerable practice. One can only wonder about the types of struggles faced by those authors who received much or most of their education in a language other than English, particularly those whose native language consist of an alphabet and grammar systems that are different from that of English (e.g., Farsi, Cantonese, Arabic), but who in a relatively short time find themselves pressured to produce manuscripts in English of equivalent quality as those produced by their native English-speaking counterparts. What are such authors to do when confronted with highly complex, technical text? Obviously, the reuse of others’ text without attribution, except for the limited reuse of technical phrases that describe a methodology of prior research (see ORI’s definition [3]), constitutes plagiarism and is highly problematic. But, what is the harm in reusing longer text strings at the level of sentences or even short paragraphs of our previously disseminated prose, even if it is as little as the 10% suggested by Moskovitz and others?

One problem I see with establishing a numerical threshold for reuse is that it may signal to authors that reuse of any type of content and from any section of a paper is always acceptable as long as it does not exceed the minimum accepted threshold. A mere percent similarity score does not distinguish between recycled technical phrases that must be reused because of their unique meaning and lack of adequate substitutes versus entire sentences or paragraphs of text that can be more easily repackaged in new language. In addition, a similarity score of, say, 20% may consist entirely of recycled technical text mainly at the phrase level whereas a 10% score may consist of one entire paragraph of an easier to rewrite literature review or discussion. Should both of these instances of recycling be equally acceptable? Blind adherence to a percent threshold may also conceivably lead some editors to reject papers automatically and overlook an author’s attempt at transparency through unconventional ways, such as that proposed by the APA Manual [19]. For example, an author may reuse large segments of a Methods section without quotations, but preface such reuse by a statement such as “we used the exact method employed in our original study and it is reproduced verbatim as follows”.^1^ To my knowledge, current versions of plagiarism detection software do not take any of these subtle issues into account. However, such author alerts about recycled text should be deemed acceptable as they satisfy the conditions of the implicit reader-writer contract even if they do not conform to the traditional mechanism of quotation and citation [5 guideline 11]. Rather than suggesting arbitrary thresholds for covert text recycling, I prefer to emphasize the spirit of transparency advocated by the reader-writer contract but I also encourage editors to be flexible in determining the appropriateness of each instance of recycled text by considering each case based on its unique circumstances (e.g., technical complexity of the questionable text and language proficiency of the author).

Moskovitz argues that rules on source attribution that have been traditionally grounded in the humanities are not equally applicable to all contexts and genres. He cites the disciplines of journalism and business as examples of disciplines in which the practice of source attribution is different and, as an example, adds that many college course syllabi often reproduce their institution’s plagiarism policy verbatim without quotation marks and attribution. However, the current discussion centers on text recycling within the context of the health sciences literature. Also, the extent to which the question of whether writing guides in the sciences provide coverage of this specific issue is, to my knowledge, unknown. But, earlier evidence indicates that writing guides across various disciplines, including the sciences, tend to do so and although there are stylistic differences in how sources are cited and how attribution is given (APA style, AMA style), all such guidance seems to be grounded in the principles of quotation and attribution common to the humanities [20].

## Conclusions

In sum, at a time when the public trust in science seems to be steadily eroding with each article that is retracted for research misconduct or for other egregious ethical research and writing lapses, a message to authors that fails to convey the need for full transparency is not in anyone’s best interests. Instituting thresholds for a minimal amount of covert text recycling may convey a message that a journal checks for text recycling and plagiarism, but adherence to an arbitrary amount of recycling without further specifying the nature and/or conditions of the reuse is, in my view, misguided. Thoughtful application of traditional rules of quotation and attribution has served the scholarly and scientific worlds well through the decades and should not be abandoned. As such, any text recycling should be practiced within the constraints outlined above. I encourage editors to heed Kleiner’s advice [21] that a certain degree of flexibility should be exercised in handling instances in which authors not dominant in English engage in inappropriate amounts of reuse, a point that I have also made in the past [22]. But, I also agree with Kleiner that such abuse of scholarly etiquette should not be tolerated in those who should know better.

## Footnotes

1 I consider the use of author alerts, such as the one described above and suggested by the APA Manual as perfectly acceptable, though it should be noted that this suggested mechanism was proposed in the most recent version of the Manual, which was published in 2010 after the publication of the 2006 version of the plagiarism document [2].
